# Tanshinone IIA and sodium tanshinone IIA sulfonate in ischemic stroke: evidence, exposure, and translational readiness

**DOI:** 10.3389/fnmol.2026.1959038

**Published:** 2026-09-23

**Authors:** Aiyue Fan, Weiting Chen, Min Tang, Yangtian Ye, Yaling Jin

**Affiliations:** 1 Department of Neurology, The First People's Hospital of Linhai, Taizhou, Zhejiang, China; 2 Department of Emergency and Intensive Care Medicine, The First People’s Hospital of Linhai, Taizhou, Zhejiang, China; 3 Department of Clinical Medicine, Hangzhou Medical College, Hangzhou, Zhejiang, China

**Keywords:** blood–brain barrier, ischemic stroke, neurovascular unit, pharmacokinetics, sodium tanshinone IIA sulfonate, tanshinone IIA, translational readiness

## Abstract

Ischemic stroke remains a major cause of death and long-term disability despite reperfusion therapy. Tanshinone IIA (Tan IIA), a lipophilic diterpene quinone, and sodium tanshinone IIA sulfonate (STS), its water-soluble sulfonated derivative, show broad activity in experimental cerebral ischemia. They are often treated as interchangeable despite differences in formulation and exposure. We critically reviewed representative pharmacological, preclinical, formulation, and clinical evidence through 28 July 2026. We distinguished active entity, formulation, causal support, replication, exposure linkage, treatment window, and patient-centered relevance. Reported effects span oxidative and mitochondrial injury, endothelial barrier failure, neuroinflammation, regulated cell death, metabolism, and repair. Most pathway claims, however, rely on single laboratories, early dosing, young healthy rodents, short follow-up, or surrogate outcomes. Human evidence comprises one 42-patient STS-plus-rt-PA trial and a 2025 meta-analysis of 22 trials involving 2,289 participants. These findings suggest short-term signals but do not establish benefits for ordinal disability, survival, recurrent stroke, or uncommon bleeding. An injectable product and human plasma pharmacokinetic methods make STS more operationally tractable; superior efficacy remains unproven. Progression should require a fixed product, exposure-linked target engagement, durable benefit in aged and comorbid reperfusion models, and independent multicenter validation. Biological plausibility remains substantially stronger than translational readiness.

## Highlights

- Tan IIA, STS, and carrier products are distinct drug products and should not be pooled without a pharmacological bridge.
- Mechanistic breadth spans the neurovascular unit, but most specific pathways lack independent replication and exposure linkage.
- The 42-patient STS-plus-rt-PA trial provides a hypothesis-generating signal, not confirmatory disability or bleeding evidence.
- STS is more operationally tractable than unmodified Tan IIA, but clinical efficacy superiority is unproven.
- Progression requires a fixed product, compartment-specific target engagement, realistic reperfusion models, and stage-gated go/no-go criteria.

## 1 Introduction

Stroke is a leading cause of death and disability worldwide. Global Burden of Disease 2021 data show rising total stroke cases despite declining age-standardized rates in many regions. Population growth, aging, and persistent prevention gaps contribute to this increase (GBD 2021 Stroke Risk Factor Collaborators, 2024). Intravenous thrombolysis and endovascular thrombectomy have transformed acute ischemic stroke treatment. The 2026 American Heart Association/American Stroke Association guideline summarizes expanding evidence for patient selection and reperfusion therapy (Prabhakaran et al., 2026). Nevertheless, reperfusion does not prevent all injury from cerebral ischemia. Many patients are ineligible, present outside the treatment window, experience incomplete arterial reopening or tissue blood-flow restoration, or develop injury after reopening. Here, recanalization denotes reopening the blocked artery, whereas tissue reperfusion denotes restoring blood flow to the affected brain. This residual burden supports investigating treatments that complement reperfusion therapy.

Repeated failures of neuroprotective drugs in clinical trials show that biological plausibility alone is insufficient (Fisher et al., 2009; Bahr-Hosseini et al., 2022; Wechsler et al., 2023; Lyden et al., 2022; Morais et al., 2024; Morais et al., 2023). Cerebral ischemia activates interacting processes that evolve over time. Energy failure and excitotoxicity begin within minutes. Oxidative and nitrosative stress, mitochondrial injury, endothelial activation, and innate immune signaling increase during early reperfusion. Blood–brain barrier disruption, edema, and regulated cell death develop over hours to days. Later, vascular remodeling, neurogenesis, synaptic plasticity, and glial responses influence recovery (Campbell et al., 2019). These phases frame the product-specific Tan IIA and STS findings evaluated below (Ou et al., 2026; Yang et al., 2011; Wang L. et al., 2020; Yu et al., 2026; Yu et al., 2025; Zhong et al., 2026). Interpreting a multi-target profile becomes difficult when each biomarker change is presented as a distinct therapeutic mechanism.

Tan IIA and STS warrant careful translational assessment as candidates addressing this unmet need. Tan IIA is a lipophilic, abietane-type diterpene quinone isolated from *Salvia miltiorrhiza* Bunge (Danshen) roots. Studies in rodent MCAO and MCAO/reperfusion models and OGD/R systems report antioxidant, anti-inflammatory, anti-apoptotic, vascular, and metabolic effects (Yang et al., 2011; Yu et al., 2026; Wang J. et al., 2020; Cai et al., 2017; Tang et al., 2010; Wang et al., 2014; Zhou et al., 2025). STS is a water-soluble derivative developed for injection and is used more frequently in clinical studies. Publications often conflate these entities, although chemical modification changes solubility, distribution, systemic exposure, blood–brain barrier transport, dose requirements, and possibly target engagement (Zhou et al., 2019; Qin et al., 2016; Mao et al., 2007; Chen et al., 2007). A mechanism observed in cultured neurons at high Tan IIA concentrations cannot automatically be attributed to intravenous STS in patients.

Earlier reviews summarized pathways through which Tan IIA may reduce cerebral ischemia–reperfusion injury (Arefnezhad et al., 2024; Guo et al., 2026; Park, 2026). They did not consistently distinguish Tan IIA from STS and carrier products, relate dose to tissue exposure, or define criteria for translational progression. We ask whether the evidence supports advancing a chemically and pharmaceutically defined Tan IIA- or STS-based drug product into confirmatory development. We distinguish product identity and exposure, map hypotheses to injury phase and neurovascular compartment, assess preclinical robustness and human evidence, and propose progression criteria. This review does not support off-label substitution for evidence-based stroke care.

Recent mechanistic and formulation reviews leave four development questions unresolved (Arefnezhad et al., 2024; Guo et al., 2026; Park, 2026). Which chemically defined product is being tested? Does a clinically deployable dose reach the compartment required by the proposed mechanism? Are findings causal, independently replicated, and obtained within a clinically realistic treatment window? What evidence justifies progression to confirmatory testing of patient-centered outcomes? We address these questions by distinguishing products and parenchymal, luminal neurovascular, and peripheral exposure, then assessing causal support and replication. We translate the resulting gaps into explicit go/no-go criteria (Table 1). This approach may inform other natural-product programs that equate shared botanical origin with pharmacological equivalence.

**Table 1:** Comparison with selected recent reviews.

Review dimension	Selected recent review emphasis	Present review	Why it matters
Drug identity	Often combines Tan IIA mechanisms or multiple tanshinones often summarized together	Separates Tan IIA, STS, and carrier-specific products	Prevents invalid transfer of mechanisms, doses, and clinical claims
Exposure	Limited product-specific integration of PK and brain exposure	Distinguishes plasma, luminal neurovascular, parenchymal, and peripheral exposure	Links mechanisms to a clinically achievable compartment-specific exposure
Evidence appraisal	General focus on mechanistic breadth	Causal support, independent replication, treatment realism, and patient-centered relevance	Separates biological plausibility from translational readiness
Clinical evidence	General summaries of small clinical studies	Reports positive and negative outcomes, subset analyses, multiplicity, and safety limitations	Reduces overinterpretation of preliminary signals
Development decision	General calls for further research	Explicit, stage-gated go/no-go criteria	Defines a testable path to phase IIb

STS, sodium tanshinone IIA sulfonate; Tan IIA, tanshinone IIA.

## 2 Scope and literature-search approach

We conducted a structured, purpose-driven search for this critical narrative review and updated it through 28 July 2026. PubMed/MEDLINE searches covered four modules: stroke efficacy and mechanism; pharmacokinetics, biodistribution, and brain penetration; formulation and delivery; and controlled human evidence and evidence syntheses. Compound terms included tanshinone IIA, tanshinone 2A, sodium tanshinone IIA sulfonate, tanshinone IIA sodium sulfonate, and relevant product names. Stroke terms included ischemic stroke, cerebral infarction, cerebral ischemia, MCAO, ischemia–reperfusion, and oxygen–glucose deprivation. We searched ClinicalTrials.gov and the Chinese Clinical Trial Registry for stroke studies. Backward and forward citation tracking covered the principal clinical, pharmacokinetic, large-animal, and mechanistic reports. Supplementary Table S3 records the search modules and their limitations.

We selected evidence to inform development decisions, without seeking an exhaustive efficacy dataset. We prioritized primary reports identifying the active entity and formulation, with interpretable dose, route, timing, model or population, functional outcome, safety, or exposure data. Causal-interruption evidence denotes experimental pathway perturbation, such as genetic manipulation or selective inhibition, testing whether the pathway is required for the reported effect. Treatment realism denotes dosing after stroke onset or reperfusion through a clinically feasible route within an emergency-care window. Appraisal considered product identity, causal support, independent replication, clinically achievable exposure, treatment realism, long-term function, and relevance to current reperfusion care. ARRIVE 2.0, STAIR, PRIMED^2^, and SPAN principles informed appraisal (Fisher et al., 2009; Bahr-Hosseini et al., 2022; Wechsler et al., 2023; Lyden et al., 2022; Morais et al., 2024; Morais et al., 2023; Percie du Sert et al., 2020). We assigned neither formal numerical PRIMED^2^ scores nor *de novo* Grading of Recommendations Assessment, Development and Evaluation (GRADE) scores. Supplementary Tables S1–S4 transparently distinguish extracted and missing information, author-assessed confidence, and the strongest permitted interpretation.

### 2.1 Interpretive classification of the evidence

We used three descriptive categories to distinguish mechanistic findings by robustness. Repeated preclinical patterns were directionally consistent findings from at least two independent studies across more than one model and/or laboratory. Repetition within one laboratory was supportive but did not constitute independent replication. Consistency supports biological plausibility without establishing clinical efficacy or translational readiness. Emerging mechanisms were recently proposed pathways supported mainly by one study with genetic, pharmacological, or other causal-interruption evidence. These hypothesis-generating findings require independent replication, clinically realistic models, and linkage to achievable exposure. Human clinical signals comprised controlled clinical findings or pooled clinical evidence. They justified further investigation only when studies had adequate methodological rigor and assessed clinically meaningful, patient-centered outcomes. These interpretive categories are not a formal grading system or equivalents of GRADE, PRIMED^2^, or regulatory qualification.

## 3 Pharmacological identity: Tan IIA is not STS

### 3.1 Chemical and formulation differences

Tan IIA is a lipophilic abietane-type diterpene quinone (C19H18O3; molecular mass 294.34 g/mol). STS is a sulfonated sodium derivative (C19H17NaO6S; molecular mass 396.39 g/mol) developed to improve aqueous solubility and intravenous compatibility (Zhou et al., 2019). Figure 1 depicts the PubChem structures for Tan IIA (compound identifier (CID) 164676) and STS (CID 23669322), including quinone groups and sulfonate connectivity. Sulfonation changes charge, ionization, membrane transport, protein binding, and potentially cellular uptake; STS is therefore chemically distinct from Tan IIA dissolved in a vehicle. Although the entities share a scaffold and may share downstream effects, pharmacological equivalence requires demonstration.

**Figure 1:**
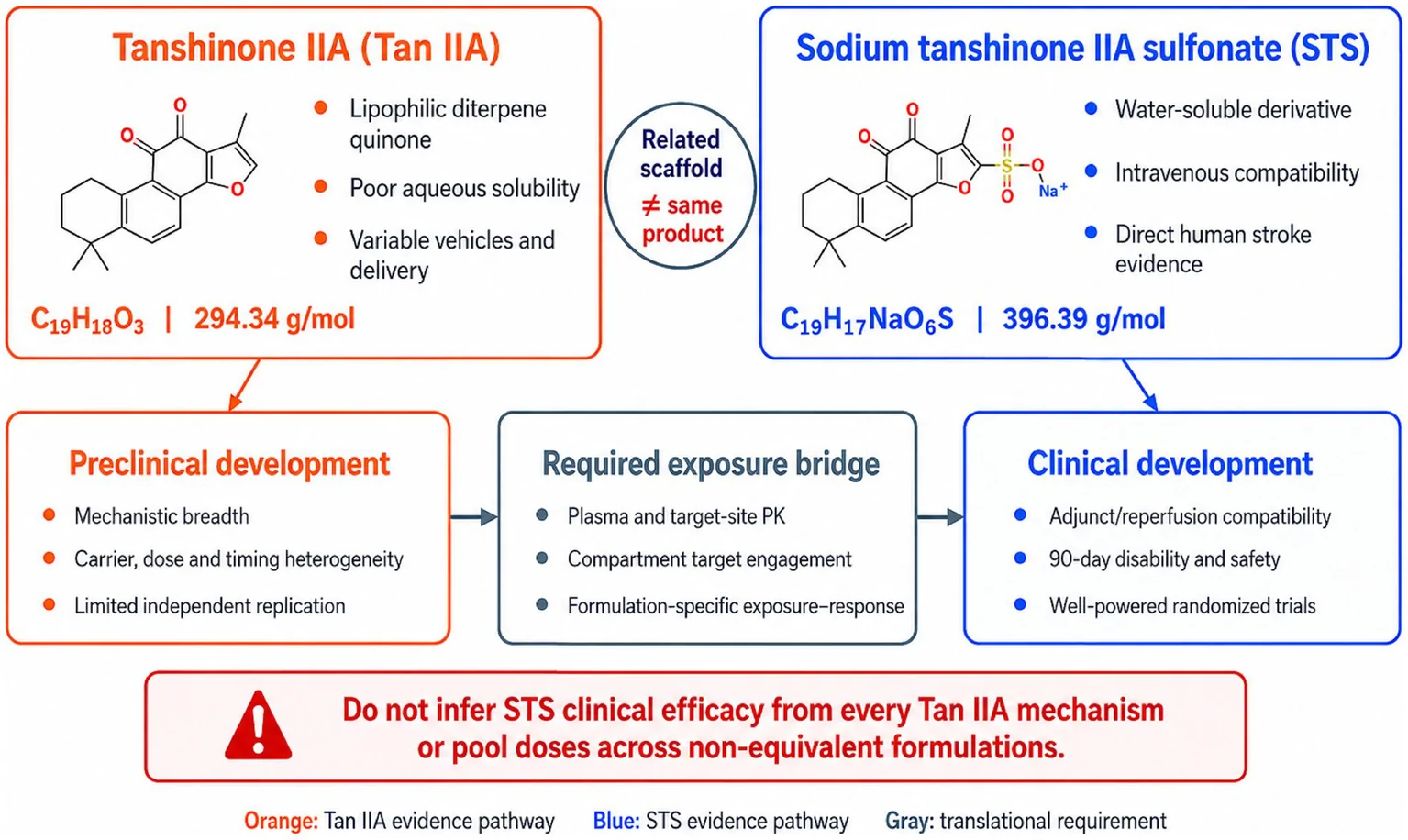
Pharmacological identity determines the evidence pathway. Tan IIA is the lipophilic parent compound (C19H18O3; 294.34 g/mol). STS is its water-soluble sulfonated sodium derivative (C19H17NaO6S; 396.39 g/mol), used in most human studies. Both structures were redrawn completely from authoritative two-dimensional PubChem records (CID 164676 and CID 23669322). Atom count, bond order, charge, sulfonate connectivity, and valence were checked before export. Mechanisms, doses, routes, and target compartments require interpretation within each product’s exposure pathway.

Preclinical studies use unmodified Tan IIA in organic solvents, aqueous STS, liposomes, polymer nanoparticles, and borneol-modified systems. Oral, intraperitoneal, intravenous, and intracisternal routes differ in absorption, distribution, peak concentration, residence time, and target-compartment exposure. Pooling nominal doses across non-equivalent products and routes therefore requires pharmacokinetic bridging. Here, active entity denotes Tan IIA or STS, and formulation denotes the delivery form. A candidate drug product comprises the fixed active entity, excipients, dose, route, and manufacturing standard intended for development. Each product follows a distinct evidence pathway (Figure 1). Clinical extrapolation requires linking mechanisms to formulation, route, exposure, and target compartment (Ou et al., 2026; Zhou et al., 2019; Ye et al., 2021; Waters et al., 2021; Kaiser et al., 2022).

### 3.2 Pharmacokinetics and brain exposure

Translation requires a clinically deployable product to reach the proposed target compartment at the necessary time and concentration. Three exposure domains must be distinguished. Parenchymal target engagement requires pharmacologically active exposure in neurons or glia; experimental evidence suggests that P-glycoprotein and other efflux processes restrict Tan IIA brain penetration (Chen et al., 2007). Luminal neurovascular engagement may occur at brain endothelial cells or circulating leukocyte-endothelial interfaces without high whole-brain concentrations. Peripheral engagement may alter hemostasis, inflammation, blood pressure, metabolism, or drug interactions. Limited brain penetration therefore does not by itself invalidate an endothelial hypothesis. It does, however, weaken direct neuronal or microglial claims unless unbound tissue exposure is demonstrated.

Sensitive LC–MS/MS methods quantify STS in human plasma, and healthy-volunteer studies show rapid systemic handling after intravenous administration (Qin et al., 2016). Animal studies also indicate rapid distribution and elimination (Mao et al., 2007). Stroke-specific plasma pharmacokinetics, free concentrations in relevant neurovascular compartments, and exposure-linked target engagement remain undefined. Total brain homogenate concentrations cannot substitute for unbound extracellular or cellular exposure. Micromolar cell-culture concentrations, nominal milligram-per-kilogram animal doses, and fixed clinical infusions therefore lack a common exposure-response scale.

### 3.3 Delivery technologies: promise and added complexity

Lipid and polymer formulations aim to improve solubility, circulation time, and brain delivery. Borneol-modified Tan IIA liposomes were associated with greater brain delivery, smaller infarcts, and lower nuclear factor-κB (NF-κB) and intercellular adhesion molecule 1 (ICAM-1) expression in rats (Ye et al., 2021). Intracisternal Tan IIA-loaded nanoparticles reduced tissue injury and functional deficits in a pig stroke model (Waters et al., 2021). A related study combined nanoparticles with neural stem cells, preventing isolation of the drug-product contribution (Kaiser et al., 2022). Reports also lack consistent carrier-only controls matched for route and composition, leaving formulation-related biological effects unresolved. These findings show that formulation can alter performance but they do not establish readiness for routine emergency stroke care.

Advanced formulations require assessment of particle size, drug loading, sterility, stability, carrier clearance, immunogenicity, device compatibility, scale-up, and regulatory classification. Intracisternal administration is unlikely to suit routine hyperacute stroke care. Brain-targeted nanoparticles may be useful, but additional exposure must be demonstrated against a matched active-drug exposure and justify the manufacturing and operational burden. An aqueous intravenous product and human plasma assays make STS easier to investigate in the near term. This feasibility advantage establishes neither superior efficacy nor reproduction of mechanisms reported for unmodified Tan IIA.

A 2026 study of 1,2-distearoyl-sn-glycero-3-phosphoethanolamine-polyethylene glycol 2000 (DSPE-PEG2000)-modified Tan IIA nanoliposomes illustrates the attribution problem (Ou et al., 2026). It evaluated a composite product without an adequately characterized biologically active carrier-only comparator, precluding automatic attribution to unmodified Tan IIA. Comparative studies should include matched vehicle and carrier-only controls, quantify drug loading and release, and measure matched active-drug exposure. They should also establish whether the carrier alters distribution or immune responses. Section 4.2 discusses the associated PGK1/Nrf2 findings. Pooling nominal doses or mechanisms across products requires this pharmacological bridge (Table 2).

**Table 2:** Product-specific pharmacology and translation of Tan IIA and STS.

Domain	Tan IIA	STS	Evidence status	Translational implication
Chemical identity	C19H18O3; approximately 294.34 g/mol; lipophilic parent compound	C19H17NaO6S; approximately 396.39 g/mol; sulfonated sodium derivative	Chemically related but non-equivalent (Zhou et al., 2019)	Do not pool products without a pharmacological bridge
Typical evidence	Predominantly cell and animal studies; several carrier products	Animal evidence and most direct human stroke evidence	Evidence pathways overlap only partially	A Tan IIA mechanism cannot be assigned automatically to intravenous STS
Formulation and route	Poor aqueous solubility; oral, intraperitoneal, intravenous carrier, or intracisternal approaches	Aqueous intravenous administration is feasible	Route and excipients materially change exposure	Fix the candidate drug product before confirmatory testing
Human pharmacokinetics	No mature PK package for a stroke-compatible product	Human plasma LC–MS/MS methods and healthy-volunteer PK are available (Qin et al., 2016)	Stroke-specific PK remains absent	Operational advantage for STS; superior efficacy unproven
Relevant exposure	Parenchymal claims require unbound neuronal/glial exposure; P-glycoprotein may restrict penetration (Chen et al., 2007)	Endothelial luminal exposure may be possible without high whole-brain concentrations	Compartment-specific target engagement is undefined	Match exposure measurement to the proposed mechanism
Human brain exposure	Not established for a deployable product	Not established	Total brain concentration is not equivalent to unbound target-site exposure	Do not extrapolate from cell concentrations or nominal animal doses
Development risk	CMC, carrier comparability, sterility, stability, scale-up, and emergency workflow	Uncertain efficacy and interactions with thrombolytics and antithrombotics; bleeding risk	Different dominant risks	Select the product and indication before efficacy confirmation

BBB, blood–brain barrier; CMC, chemistry, manufacturing and controls; LC–MS/MS, liquid chromatography–tandem mass spectrometry; PK, pharmacokinetics; STS, sodium tanshinone IIA sulfonate; Tan IIA, tanshinone IIA.

## 4 Phase-resolved mechanisms across the neurovascular unit

The neurovascular unit comprises neurons, brain endothelial cells, pericytes, astrocytes, microglia, extracellular matrix, and circulating immune cells. We organize reported actions by injury phase and assign each claim to Tan IIA, STS, a carrier-specific product, or explicitly labeled as cross-entity evidence. Phases overlap, and pathway direction may vary with time and cell type. Figure 2 presents hypotheses, distinguishing repeated preclinical patterns, emerging mechanisms, and human clinical signals without implying a unified mechanism.

**Figure 2:**
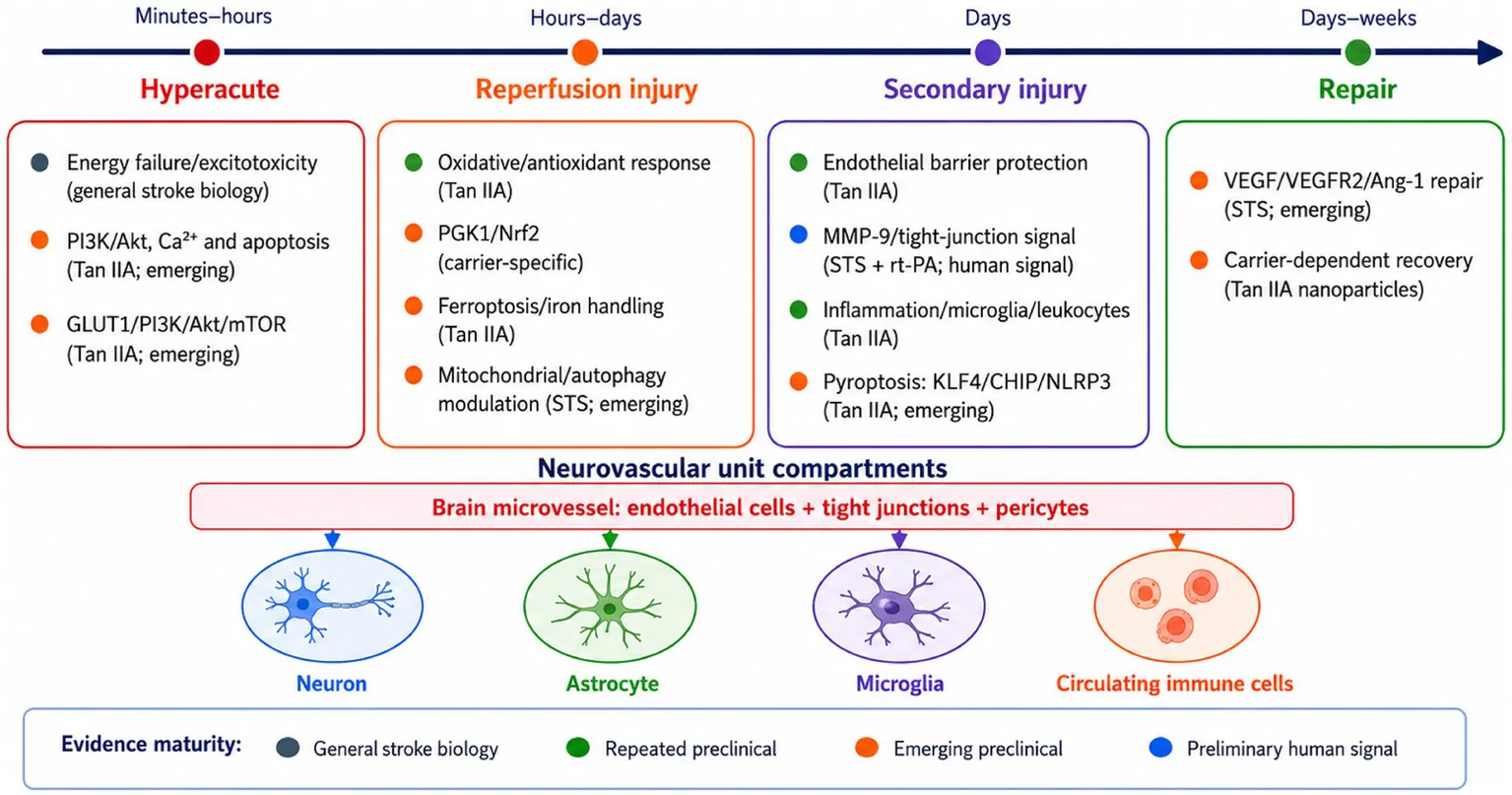
Phase-resolved, product-specific hypotheses across the neurovascular unit. Gray denotes general stroke pathophysiology; green, repeated preclinical patterns; orange, emerging or predominantly single-study mechanisms; and blue, preliminary, hypothesis-generating human clinical signals. Node-to-reference mapping is as follows: general energy failure/excitotoxicity (Campbell et al., 2019); Tan IIA PI3K/Akt-related calcium/apoptosis (Tang et al., 2012); GLUT1/PI3K/Akt/mTOR (Wang J. et al., 2020); antioxidant/Nrf2 and iron-handling/ferroptosis (Ou et al., 2026; Yang et al., 2011; Yu et al., 2025; Cai et al., 2017); endothelial-barrier effects (Tang et al., 2010; Zhang et al., 2010); inflammatory/microglial/leukocyte findings (Yu et al., 2026; Wang et al., 2014; Zhou et al., 2025; Chen et al., 2012; Song et al., 2021); and KLF4/CHIP/NLRP3-related pyroptosis (Zhong et al., 2026). STS-associated nodes comprise mitochondrial/autophagy findings (Wang L. et al., 2020; Ma et al., 2023) and VEGF/VEGFR2/Ang-1-related repair (Xu et al., 2021). Carrier-dependent porcine recovery findings concern Tan IIA nanoparticles (Waters et al., 2021; Kaiser et al., 2022); the combined neural stem-cell study cannot isolate the nanoparticle contribution to long-term recovery. The preliminary human signal concerns STS plus rt-PA and MMP-9/tight-junction-related findings (Ji et al., 2017). Colors summarize evidence maturity, not effect size or clinical efficacy. Cross-entity convergence does not imply pharmacological equivalence.

### 4.1 Hyperacute energy failure, excitotoxic stress and metabolic dysfunction

Arterial occlusion rapidly causes energy failure, membrane depolarization, ion imbalance, glutamate release, and intracellular calcium accumulation (Campbell et al., 2019). These general ischemic processes are not established Tan IIA targets. In a permanent MCAO/OGD study, Tan IIA monotherapy reduced injury, altered intracellular calcium and apoptosis-related measures, and increased Akt phosphorylation (Tang et al., 2012). PI3K inhibition abolished the Akt-associated effect in cultured neurons (Tang et al., 2012). This supports an emerging PI3K/Akt cell-survival hypothesis without establishing ATP preservation or a clinically relevant effect in reperfused patients.

An animal and cell study linked Tan IIA to glucose transporter 1 (GLUT1) and PI3K/mTOR signaling (Wang J. et al., 2020). It inadequately described preparation of poorly water-soluble Tan IIA for intravenous administration and lacked a matched formulation control. The metabolic and neuroprotective effects therefore cannot be confidently attributed to Tan IIA rather than the delivery matrix or preparation. Figure 2 classifies GLUT1/PI3K/Akt/mTOR as an emerging preclinical mechanism, without human clinical evidence. Replication should specify solvents and excipients, include matched vehicle controls, measure exposure, and combine metabolic-flux assays with cerebral microdialysis, or imaging.

### 4.2 Oxidative stress and mitochondrial dysfunction

Reperfusion can cause oxidative and mitochondrial injury through reactive oxygen species from damaged mitochondria, xanthine oxidase, NADPH oxidases, and inflammatory cells. Tan IIA has been associated with lower malondialdehyde and reactive oxygen species, preserved antioxidant activity, and reduced neuronal injury (Yang et al., 2011; Cai et al., 2017). Cai et al. linked protection to nuclear factor erythroid 2-related factor 2 (Nrf2) activation (Cai et al., 2017). However, they described intraperitoneal Tan IIA prepared in phosphate-buffered saline despite its limited aqueous solubility, without sufficient solubilization detail. The administered preparation and Tan IIA contribution therefore remain uncertain. A carrier-specific 2026 study associated Tan IIA nanoliposomes with phosphoglycerate kinase 1 (PGK1)/Nrf2 activation (Ou et al., 2026). These reports support an antioxidant hypothesis, but formulation uncertainty and entity specificity preclude direct extrapolation to intravenous STS.

Ferroptosis is part of oxidative injury driven by iron-dependent lipid peroxidation. Tan IIA altered brain iron-handling proteins in an earlier study (Yang et al., 2011). A 2025 report linked reduced neuronal ferroptosis to the miR-449a/ACSL4 pathway (Yu et al., 2025). These early findings derive mainly from single laboratories. Confirmation requires orthogonal ferroptosis assays, causal perturbation, matched formulation controls, delayed treatment, and measured brain exposure. Lower oxidative markers may reflect smaller infarcts rather than cause protection. Target engagement must therefore be demonstrated at clinically achievable exposure after imaging or reperfusion.

In a microglia–neuron co-culture OGD/R model, STS increased microglial autophagy and was associated with neuronal mitochondrial protection (Ma et al., 2023), whereas an *in vivo* cerebral ischemia–reperfusion study linked STS to inhibition of excessive autophagy and inflammation (Wang L. et al., 2020). These context-dependent findings should not be treated as mechanistically contradictory; autophagic-flux interpretation and validation requirements are discussed in Section 4.5.

### 4.3 Blood–brain barrier and microvascular protection

Blood–brain barrier disruption contributes to vasogenic edema, hemorrhagic transformation, and poor outcomes after reperfusion, making it relevant to adjunctive treatment. In transient middle cerebral artery occlusion, Tan IIA reduced Evans blue leakage, edema, ICAM-1, and matrix metalloproteinase-9 (MMP-9), while preserving ZO-1 and occludin (Tang et al., 2010). A human brain microvascular endothelial barrier model also suggested protection against leukocyte-associated hypoxia-reoxygenation injury (Zhang et al., 2010). Together, these findings support a direct endothelial contribution beyond purely neuronal effects.

Endothelial barrier protection is the most coherent cross-domain hypothesis, but remains unvalidated as a mediator of clinical benefit. In the small STS-plus-rt-PA trial, treatment was associated with day-10 changes in MMP-9, tissue inhibitor of metalloproteinase 1 (TIMP-1), claudin-5, and CT-perfusion permeability (Ji et al., 2017). These analyses included a 30-patient imaging/biomarker subset, classified in Figure 2 as a preliminary, hypothesis-generating human signal. Convergence with Tan IIA endothelial and rodent findings crosses pharmacological entities and does not establish a shared exposure pathway in patients (Tang et al., 2010; Zhang et al., 2010). Lower circulating tight-junction-related markers do not directly establish structural barrier preservation. No formal mediation analysis linked barrier changes to disability outcomes.

Barrier protection could improve reperfusion safety or reduce edema after successful recanalization. Prolonged treatment might, however, impair beneficial immune-cell entry or vascular repair. Endothelium stabilization during the first 24–72 h differs biologically from suppressing vascular remodeling during recovery. Dose and duration should therefore match the stage of stroke rather than follow cardiovascular regimens.

### 4.4 Neuroinflammation and glial responses

Ischemic tissue releases danger signals that activate microglia, astrocytes, endothelial cells, and infiltrating leukocytes (Campbell et al., 2019). Tan IIA-associated patterns include reduced high-mobility group box 1 (HMGB1), NF-κB activation, glial fibrillary acidic protein, inflammatory cytokines, and apoptosis after transient focal ischemia (Wang et al., 2014). Other studies reported reduced macrophage migration inhibitory factor, cytokine release, and neutrophil infiltration (Chen et al., 2012), or NF-κB-linked changes in microglial activation and polarization (Song et al., 2021). Toll-like receptor 4 (TLR4)/NF-κB and transglutaminase 2/pannexin 1 (TGM2/PANX1) hypotheses remain predominantly single-study mechanisms (Yu et al., 2026; Zhou et al., 2025). Pyroptosis is considered here as an inflammatory cell-death program. A 2026 report associated Tan IIA with Krüppel-like factor 4/CHIP/NLRP3 signaling, but direct causal testing in animals was incomplete (Zhong et al., 2026).

Post-stroke inflammation can have both detrimental and beneficial effects. Early innate responses remove debris and may limit injury; later immune and glial responses influence angiogenesis, remyelination, and synaptic repair. Broad suppression of NF-κB or leukocyte entry may therefore help at one stage and harm at another. Studies should distinguish resident microglia from infiltrating monocytes, use single-cell or spatial methods when feasible, and relate immune-cell states to long-term recovery. Lower tumor necrosis factor-α and interleukin-1β concentrations at 24 h alone cannot define an effective immune treatment strategy.

Stroke-related immune suppression contributes to pneumonia and other complications, making infection risk relevant to development. Tan IIA and STS studies rarely assess systemic immune function, bacterial clearance, or infection outcomes. Acute stroke candidates should therefore be tested for both brain anti-inflammatory effects and systemic immune effects. This is especially relevant to older patients with dysphagia, aspiration risk, or invasive procedures.

### 4.5 Regulated cell death and autophagy

Apoptosis is the regulated cell-death pathway most frequently reported in Tan IIA studies. Tan IIA monotherapy altered Bcl-2, Bax, caspase-3, and TUNEL-related measures in a permanent MCAO/OGD study (Tang et al., 2012), with overlapping findings in other stroke studies (Wang et al., 2014; Song et al., 2021). These short-term markers do not establish apoptosis as the dominant cause of neuronal loss.

Autophagy should be interpreted as dynamic flux rather than as a static protein signature. In a microglia–neuron co-culture OGD/R model, STS increased microglial autophagy and was associated with neuronal mitochondrial protection (Ma et al., 2023), whereas an *in vivo* cerebral ischemia–reperfusion study linked STS to inhibition of excessive autophagy and inflammation (Wang L. et al., 2020). The 2023 co-culture study assessed microglial autophagy-related changes, but *in vivo* cell-specific flux, product-specific exposure linkage, and durable functional confirmation remain lacking.

Ferroptosis and pyroptosis are addressed in Sections 4.2 and 4.4, respectively. Confirmation of apoptosis and autophagic flux requires cell-specific sampling, orthogonal death assays, appropriate lysosomal inhibition, *in vivo* causal perturbation, and long-term functional outcomes. Emerging mechanisms should inform clinical biomarkers or continuation decisions only after independent replication with orthogonal assays and causal interruption *in vivo* (Ou et al., 2026; Yu et al., 2026; Yu et al., 2025; Zhong et al., 2026; Zhou et al., 2025). Validation must also use realistic treatment delays, matched vehicle or carrier controls, and measured product-specific exposure.

### 4.6 Subacute repair, angiogenesis and functional recovery

After the first few days, therapeutic goals shift from tissue protection toward vascular remodeling, neurogenesis, axonal growth, and functional recovery. One STS study reported increased VEGF, VEGFR2, platelet endothelial cell adhesion molecule 1 (CD31), and angiopoietin-1 alongside improved experimental outcomes (Xu et al., 2021). Carrier-based porcine studies reported tissue protection and functional recovery. However, the combination study could not isolate Tan IIA nanoparticles from neural stem-cell therapy (Waters et al., 2021; Kaiser et al., 2022). Figure 2 therefore classifies VEGF/VEGFR2/Ang-1-related repair as an emerging product-specific preclinical mechanism, without replication or clinical confirmation.

Early barrier stabilization and later angiogenesis may require different products, schedules, and outcomes. VEGF can support vascular repair but may increase permeability when expressed too early or at high levels. The STS angiogenesis report (Xu et al., 2021) is therefore a product-specific preclinical signal that cannot support a treatment schedule. Separate validation should define exposure, treatment onset, duration, and long-term motor, sensory, cognitive, white-matter, and network outcomes. 24-h infarct volume alone is insufficient for assessing these effects.

## 5 Robustness and translational readiness of the preclinical evidence

Directionally consistent experimental findings support biological plausibility, and recent reports add candidate pathways and delivery systems (Ou et al., 2026; Yu et al., 2026; Zhong et al., 2026; Zhou et al., 2025). Translational readiness, however, depends on product definition, exposure, design quality, and reproducibility. A 2023 meta-analysis of *Salvia miltiorrhiza*-related animal treatments reported smaller infarcts and improved neurological or biomarker outcomes (Xie et al., 2023). The synthesis pooled preclinical Tanshinone IIA-labeled studies but did not establish efficacy for a single fixed, clinically deployable Tan IIA or STS drug product (Xie et al., 2023). Pooling also cannot remove bias from flexible analyses, incomplete blinding, or selective publication in small studies. A pilot synthesis of female-rodent neuroprotection studies associated low quality and unreported blinding or random allocation with larger or less reliable effects (Gunderson et al., 2024). Mechanistic breadth does not establish a replicated development hypothesis.

### 5.1 Population mismatch

Most Tan IIA stroke studies use young adult male rodents without hypertension, diabetes, atrial fibrillation, kidney disease, atherosclerosis, or prior antithrombotic treatment. Patients are typically older, include both sexes, and often have several of these conditions. Age affects collateral blood flow, blood–brain barrier function, immune, mitochondrial reserve, and drug clearance; diabetes and hypertension alter oxidative stress and endothelial responses. The Stroke Preclinical Assessment Network (SPAN), which included 1,789 mice followed for 1 month (Morais et al., 2024). Age, body weight, diet, anesthesia duration, and filament characteristics predicted mortality, neurological scores, behavioral outcomes, or tissue injury (Morais et al., 2024). Effects in young rodents may therefore diminish, require dose adjustment, or introduce safety concerns in older patients with comorbidities.

Confirmatory programs should include aged animals of both sexes and at least one common vascular comorbidity (Wechsler et al., 2023; Lyden et al., 2022; Morais et al., 2024). Sex should be analyzed as a biological variable, beyond numerical balance. Kidney and liver function merit assessment because they may alter STS exposure. Despite greater cost and variability, efficacy should persist across clinically relevant variation before it advances to human testing.

### 5.2 Model and treatment-window mismatch

The Intraluminal-filament middle cerebral artery occlusion model is practical but does not reproduce clot composition, thrombolysis, spontaneous embolic fragmentation, or thrombectomy. Embolic models represent thrombus-mediated occlusion and allow thrombolytic testing, although clot size and recanalization vary. Permanent occlusion models address non-recanalizing stroke but cannot assess reperfusion injury or adjunctive safety. Temporary filament occlusion controls reperfusion but introduces procedural and filament-dependent variability. Oxygen–glucose deprivation/reoxygenation isolates cellular processes without circulation, immune recruitment, or systemic pharmacokinetics. These models cannot be pooled as equivalent. In the first SPAN control cohort, filament choice, comorbidity, sex, cerebral blood-flow monitoring, and anesthesia affected performance across six laboratories (Morais et al., 2023). The small STS-plus-rt-PA trial was the principal direct adjunctive study included here; none tested tenecteplase or mechanical reperfusion (Ji et al., 2017). Adjunctive use remains the most plausible clinical role and requires reperfusion-compatible translational designs (Wechsler et al., 2023; Lyden et al., 2022).

Dosing before arterial occlusion or immediately before reperfusion can show biological activity but poorly represents emergency care. Studies should define the latest effective dosing time after symptom onset and recanalization and use imaging or physiological entry criteria. They should test dose–response relationships rather than adopt a single dose from non-stroke work. STAIR recommends reproducible dose–response findings, realistic windows, physiological monitoring, and tissue and functional outcomes (Fisher et al., 2009). SPAN shows that centralized randomization and blinding can accommodate planned biological and procedural variation across independent laboratories (Lyden et al., 2022; Morais et al., 2023).

### 5.3 Outcome and follow-up limitations

Infarct volume and early neurological scores depend on measurement methods and may not predict sustained recovery (Fisher et al., 2009; Wechsler et al., 2023; Morais et al., 2024). Edema can distort infarct measurements, and simple rodent scores have limited sensitivity. Studies ending at 24 or 72 h miss delayed inflammation, tissue loss, and compensatory recovery. A 90-day middle cerebral artery occlusion study showed that short assessments can miss delayed deficits and temporary, reversible treatment effects (Encarnacion et al., 2011). Such limitations can produce false-positive claims of neuroprotection. Development therefore requires several weeks of follow-up, prespecified behavioral tests, mortality reporting, and assessment of hemorrhagic transformation. Cognitive and emotional outcomes should be assessed when relevant to the expected effect.

### 5.4 Methodological quality and reporting

ARRIVE 2.0 specifies essential reporting items, including study design, sample-size planning, eligibility criteria, randomization, blinding, outcomes, and statistical methods (Percie du Sert et al., 2020). PRIMED^2^ evaluates cumulative preclinical evidence for an acute stroke therapy across 11 domains covering animal diversity, treatment timing and feasible windows/routes, dose–response, and the magnitude of imaging and behavioral effects (Bahr-Hosseini et al., 2022). Stroke-model reviews and multicenter programs show that absent blinding or randomization, low study quality, and unmodeled procedural variation can alter estimated effects and outcomes (Lyden et al., 2022; Morais et al., 2024; Morais et al., 2023; Gunderson et al., 2024). Despite broad mechanistic evidence, the Tan IIA and STS literature remains weak in independent replication, realistic treatment windows, exposure-linked target engagement, comorbid models, and multicenter testing (Fisher et al., 2009; Bahr-Hosseini et al., 2022; Wechsler et al., 2023).

### 5.5 The need for independent and multicenter confirmation

Many mechanistic claims come from one laboratory, model, dose, and short-term outcome. Independent replication is uncommon, and formulation details or matched vehicle controls are often inadequate (Ou et al., 2026; Wang J. et al., 2020; Cai et al., 2017). These limitations recur in intravenous Tan IIA, phosphate-buffered saline, and nanoliposome reports. SPAN demonstrates the feasibility of randomized, placebo-controlled, blinded preclinical trials across laboratories (Lyden et al., 2022; Morais et al., 2024; Morais et al., 2023). Before another efficacy trial, a selected product requires preregistered testing, chemically matched vehicle or carrier controls, measured exposure, and clinically relevant reperfusion. Independent replication should involve at least two centers. Figure 3 summarizes the progression from broad mechanistic evidence to sparse patient-centered confirmation.

**Figure 3:**
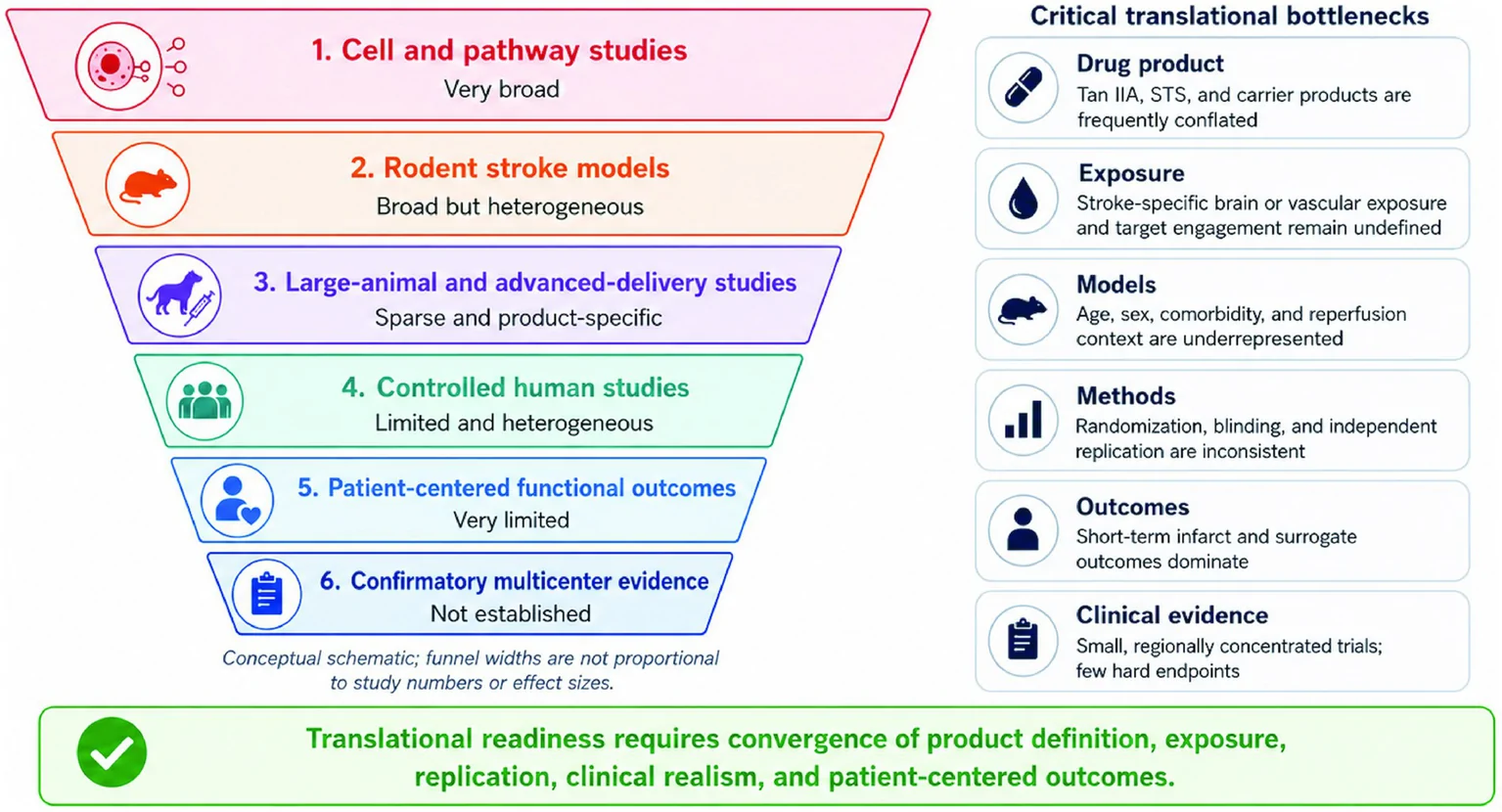
Evidence landscape and translational bottlenecks. The conceptual funnel depicts increasing requirements from mechanistic breadth to confirmatory multicenter evidence on patient-centered outcomes. Widths are not proportional to study numbers or effect sizes. Bottlenecks include product definition, exposure and target engagement, clinically realistic models, methodological rigor, independent replication, and robust disability and safety outcomes.

## 6 Human clinical evidence

### 6.1 The randomized STS-plus-thrombolysis study

The most informative controlled human study is a 2017 single-center, randomized, double-blind, placebo-controlled trial (Ji et al., 2017). Of 46 enrolled patients, 42 were analyzed after three incomplete treatment courses and one loss to follow-up. All received rt-PA and were assigned intravenous STS 60 mg/day or saline for 10 days (21 per group). At 90 days, mRS ≤ 1 occurred in 16/21 STS recipients and 9/21 placebo recipients. The risk ratio was 1.78 (95% CI 1.03–3.08), and the absolute risk difference was 33.3% (95% CI 5.4–61.3%; published *p* = 0.028). Confidence intervals were calculated from the published summary data. Day-10 NIHSS was 2.81 ± 2.64 versus 4.10 ± 3.81 (mean difference −1.29, approximate 95% CI −3.27 to 0.69; *p* = 0.211); activities of daily living were 82.62 ± 20.89 versus 72.38 ± 25.18 (mean difference 10.24, approximate 95% CI −3.75 to 24.23; *p* = 0.159). Infarct volume was 2.020 ± 1.762 versus 2.415 ± 3.083 cm^3^ (mean difference −0.40 cm^3^, approximate 95% CI −2.34 to 1.55; *p* = 0.676). CT-perfusion and biomarker analyses included only 30 patients (16 STS and 14 placebo). Day-10 MMP-9 and claudin-5 were lower and TIMP-1 was higher with STS. No formal mediation analysis established that these surrogate changes caused functional benefit.

The trial provides a hypothesis-generating signal with substantial uncertainty. Hemorrhagic transformation occurred in 2/21 STS recipients and 5/21 controls (risk ratio 0.40, 95% CI 0.09–1.84; *p* = 0.410). This imprecise estimate cannot establish safety. Limitations include the small sample, single-center design, attrition, imaging-subset analysis, mRS dichotomization, multiple clinical and biomarker comparisons, and limited power for symptomatic intracranial hemorrhage. A favorable dichotomized 90-day signal can coexist with negative early neurological, daily-living, and infarct-volume outcomes. Replication requires a multicenter trial with ordinal mRS analysis and centrally adjudicated safety.

### 6.2 Meta-analytic evidence

A 2025 systematic review and meta-analysis included 22 randomized trials and 2,289 participants, with searches updated through February 2024 (Liu et al., 2025). Pooled findings suggested improvements in short-term neurological deficit scores and several physiological, hemorheological, coagulation, lipid, and biochemical measures. Evidence relied heavily on heterogeneous intravenous regimens and nonstandard outcomes, including a composite ‘total effective rate.’ Activities of daily living did not improve significantly. The analysis provided no robust evidence for ordinal 90-day disability, survival, recurrent stroke, quality of life, or uncommon bleeding events.

Confidence is limited by small samples, poorly reported allocation concealment and blinding, variable background care, heterogeneous doses and durations, short follow-up, and regional concentration. Reporting of hard outcomes was also sparse, and pooling cannot resolve these limitations. Future trials should prioritize ordinal analysis of the full 90-day mRS distribution, with NIHSS, imaging, and biomarkers as supportive or mechanistic outcomes. The meta-analysis supports further investigation, but it does not establish readiness for guideline-supported STS use.

### 6.3 Safety and drug-interaction uncertainty

Patients with stroke often receive thrombolytics, antiplatelet drugs, anticoagulants, statins, antihypertensives, and contrast media, and many have impaired kidney or liver function. Danshen-derived compounds, including Tan IIA and STS, have reported anticoagulant, antiplatelet, vascular, and vasodilator effects (Zhou et al., 2019). These may support microcirculation but could interact with alteplase, tenecteplase, dual antiplatelet therapy, or anticoagulation. Clinical studies are too small to exclude a modest increase in intracranial or systemic bleeding, and adverse-event reporting is inconsistent. Infusion reactions, hypersensitivity, and organ toxicity also require careful monitoring.

Potential pharmacokinetic and pharmacodynamic interactions require testing before definitive efficacy trials. Non-stroke findings on cytochrome P450 enzymes and hemostasis support formal interaction studies with thrombolytics, antiplatelet agents, anticoagulants, and statins. Symptomatic intracranial hemorrhage (sICH) should be a key secondary safety outcome, defined by a prespecified recognized standard, such as ECASS or SITS-MOST, and centrally adjudicated. Trials should also assess systemic major bleeding, hemorrhagic transformation, platelet and coagulation measures, infusion reactions, and organ toxicity. Adequate follow-up and analysis stratified by exposure are also required.

### 6.4 Current clinical position

Tan IIA and STS are investigational adjuncts, without established roles in acute ischemic stroke treatment. Guideline-supported care emphasizes rapid diagnosis, reperfusion eligibility, stroke-unit management, complication prevention, and early secondary prevention (Prabhakaran et al., 2026). Neither compound is included among guideline-supported acute stroke treatments or should replace thrombolysis, thrombectomy, antithrombotic treatment, or other standard care.

The 2017 report gives the trial registration as ChiCTR-ONRC-14004659 (Ji et al., 2017). Registry confirmation of timing relative to first enrollment is required before describing registration as prospective. A ClinicalTrials.gov search through 28 July 2026 identified no clearly active ischemic-stroke development program under the principal Tan IIA or STS terms. This search-dependent finding does not exclude regional studies or studies registered under other names.

China has contributed injectable STS development and availability, regional pharmacokinetic methods, the registered STS-plus-rt-PA trial, and a substantial Chinese-language clinical literature (Zhou et al., 2019; Qin et al., 2016; Ji et al., 2017; Liu et al., 2025). Challenges include variable product naming, incomplete allocation-concealment and blinding reports, heterogeneous care and outcomes, regional concentration, and limited indexing outside Chinese databases. Evidence integration requires bilingual searches, deduplication, verification of registry timing and product identity, and translation of outcome definitions. Risk-of-bias and exposure standards should match those applied to English-language studies. Neither historical use nor publication language should change the evidentiary threshold.

## 7 Why translation remains limited

Translation depends on whether a defined drug product can reproducibly engage its target and improve patient-centered outcomes at a clinically achievable exposure. The current literature often combines Tan IIA, STS, and carriers despite physicochemical and exposure differences, with little linkage between experimental exposure and feasible clinical doses. Young healthy rodents, early or invasive dosing, and short-term surrogate outcomes predominate. Broad biomarker changes do not establish a causal, independently replicated development hypothesis. Human evidence remains geographically concentrated and underpowered for disability and uncommon harm.

These limitations recur across stroke neuroprotection programs and also constrain this review. We conducted a purpose-driven narrative audit, rather than a prospectively registered systematic review. Searches relied mainly on PubMed/MEDLINE, registries, and citation tracking; not every report underwent formal study-level risk-of-bias adjudication. Chinese-language and non-indexed evidence may be underrepresented, making the evidence matrix representative rather than exhaustive. Development should focus on one product and clinical setting, a measurable target, realistic treatment windows, and independent replication before large trials. Table 3 and Figure 4 convert these gaps into stage-gated actions.

**Table 3:** Translational barriers and minimum next actions at each stage.

Gate/barrier	Current evidence problem	Minimum next action
Product and CMC	Tan IIA, STS, and carrier products are often treated as one intervention.	Fix active entity, formulation, route, impurities, stability, release specifications, and scalable manufacturing.
Exposure and target engagement	Cell concentrations and animal doses are not linked to relevant human compartments.	Measure plasma PK and mechanism-relevant luminal or unbound parenchymal exposure; model exposure-response.
Therapeutic window and reperfusion	Pretreatment and immediate dosing predominate; evidence of compatibility with modern thrombolysis/thrombectomy is sparse.	Test delayed dosing after imaging with alteplase/tenecteplase and mechanical reperfusion; assess bleeding.
Population and model diversity	Young healthy male rodents predominate.	Use aged animals of both sexes, common vascular comorbidities, and prespecified heterogeneity.
Durable efficacy	Short-term infarct volume and simple scores dominate.	Require ≥28-day multidomain function, cognition where relevant, mortality, hemorrhagic transformation, and toxicity assessment.
Independent replication	Many product-dose-mechanism claims originate from one laboratory.	Preregister and replicate the primary hypothesis in at least two independent centers using blinded methods.
Phase Ib/IIa	Stroke-specific dose, PK, interactions, and target engagement are unclear.	Establish dose/exposure, renal-hepatic covariates, interaction safety, and centrally adjudicated events.
Phase IIb	Insufficient power for hard functional outcomes and uncommon safety events.	Use a multicenter, double-blind design; ordinal 90-day mRS; standardized sICH/major bleeding; death; and quality of life.
Phase III gate	Biomarker breadth may encourage premature progression.	Proceed only after reproducible product, exposure, target engagement, safety, and patient-centered efficacy criteria are met.

CMC, chemistry, manufacturing and controls; mRS, modified Rankin Scale; PK, pharmacokinetics; sICH, symptomatic intracranial hemorrhage.

**Figure 4:**
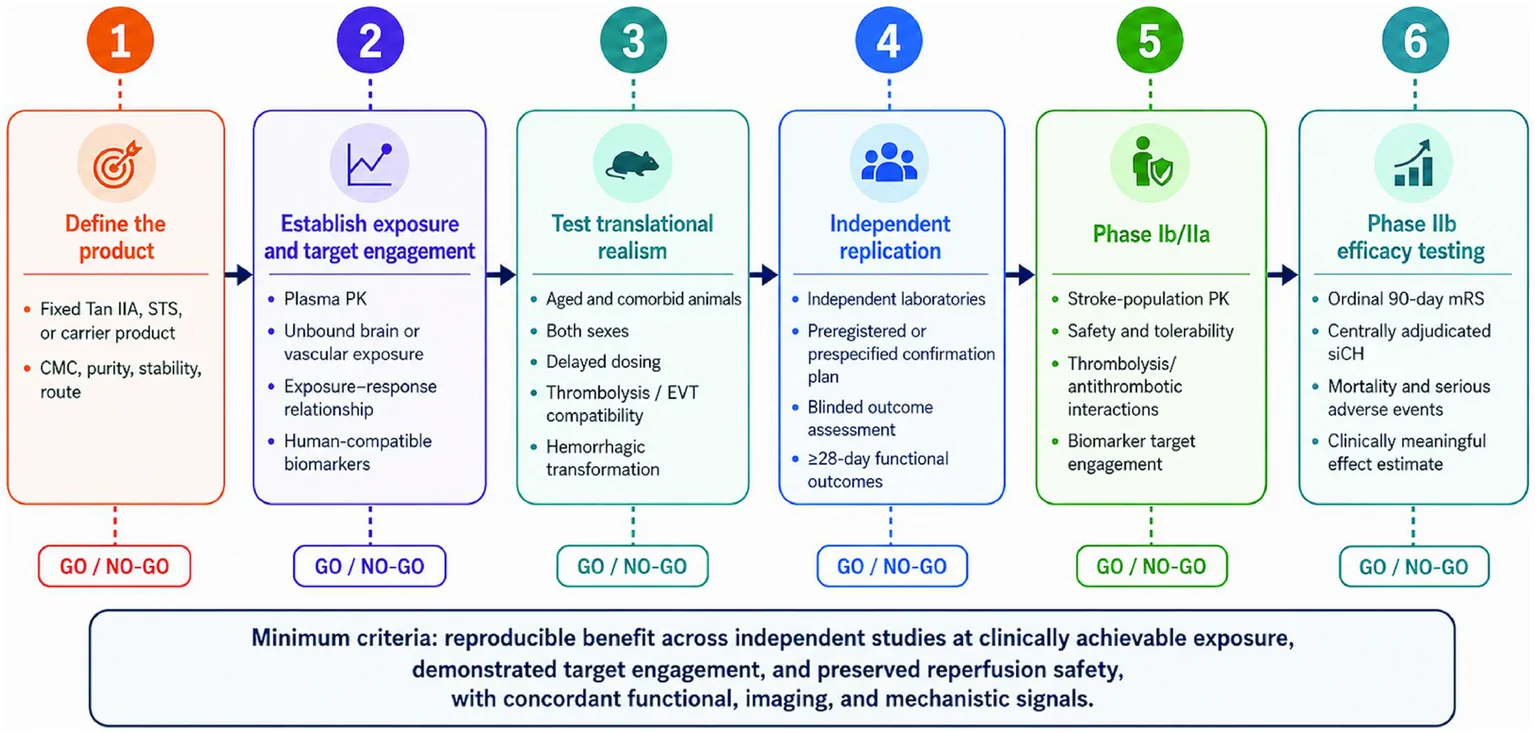
Stage-gated development roadmap for a defined tanshinone drug product. Six stages cover product definition; exposure and target engagement; translational realism, independent replication; phase Ib/IIa safety, pharmacology, and biomarkers; and phase IIb patient-centered efficacy. Translational realism requires aged, comorbid models with delayed treatment and reperfusion compatibility. Each stage has explicit go/no-go criteria; phase III requires completion of all preceding stages. Abbreviations: CMC, chemistry, manufacturing and controls; EVT, endovascular thrombectomy; mRS, modified Rankin Scale; PK, pharmacokinetics; sICH, symptomatic intracranial hemorrhage; STS, sodium tanshinone IIA sulfonate; Tan IIA, tanshinone IIA.

## 8 A translational development roadmap

### 8.1 Select the clinical product and indication

Development must first select STS, a clinically deployable formulation of unmodified Tan IIA, or a carrier product. STS has the strongest operational case because it is water soluble, measurable in human plasma, and has been tested in controlled stroke studies. This establishes neither pharmacodynamic equivalence to Tan IIA nor superior efficacy. A narrow near-term indication could target adjunctive endothelial and neurovascular protection after thrombolysis or thrombectomy in patients at substantial risk of barrier injury. Subacute repair requires separate development because mechanisms, windows, duration, and outcomes differ. Specifications, impurities, stability, and manufacturing should be fixed before confirmatory animal studies to match the intended clinical product. Figure 4 sequences product selection, exposure bridging, realistic preclinical confirmation, early human pharmacology and interaction testing, and a biomarker-informed phase II study. Each stage requires a prespecified go/no-go decision.

### 8.2 Build an exposure-linked preclinical package

A confirmatory preclinical program should include the following elements:

- Measure plasma and unbound brain pharmacokinetics after clinically practical intravenous dosing, including in aged animals and animals with comorbidities.
- Establish dose–response and treatment-window relationships after imaging-confirmed arterial occlusion and after reperfusion.
- Demonstrate target engagement with a prespecified barrier, inflammatory, or metabolic biomarker that is linked to drug exposure.
- Test interactions with alteplase or tenecteplase and model endovascular reperfusion when feasible.
- Assess hemorrhagic transformation, mortality, infection, organ toxicity, and neurological function for at least 28 days.
- Independently replicate the primary finding using preregistration, allocation concealment, and blinded analysis.
- Use large-animal or multicenter platforms only after defining the product, dose, and main mechanistic hypothesis.

Prespecified progression criteria should require fixed product and CMC specifications, clinically achievable exposure-response relationships, and target engagement in the relevant compartment. They should also require durable multidomain function with delayed, reperfusion-compatible dosing, acceptable intracranial and systemic bleeding, and independent replication in at least two centers. Development should pause when benefit is confined to pretreatment, 24-h infarct volume, clinically infeasible exposure, or an inadequately characterized carrier. These criteria follow the decision-focused principles of STAIR and related translational initiatives (Fisher et al., 2009; Bahr-Hosseini et al., 2022; Wechsler et al., 2023).

### 8.3 Early-phase clinical development

Phase Ib/IIa studies should establish stroke-specific pharmacokinetics, dose tolerance, renal and hepatic covariates, interactions with reperfusion and antithrombotic treatments, and human target engagement. Repeated hemostatic, endothelial, and inflammatory measures may support mechanism, while bleeding and neurological events require central review. Identification of an acceptable dose and exposure-target relationship should precede multicenter, randomized, double-blind phase IIb testing of preliminary efficacy in a prespecified population.

Phase IIb should prioritize a prespecified ordinal shift in 90-day mRS. Secondary outcomes should include early NIHSS change, infarct growth, edema, symptomatic intracranial hemorrhage, systemic major bleeding, death, activities of daily living, and quality of life. Dynamic contrast-enhanced MRI or validated CT-perfusion permeability, MMP-9, GFAP, and neurofilament light may support target engagement but cannot replace disability outcomes. Imaging- or biomarker-based enrichment must be defined before enrollment and reproducible across centers.

Phase III requires independent evidence of reproducible manufacturing, clinically achievable exposure, relevant target engagement, acceptable hemorrhagic and systemic safety, and a credible patient-centered efficacy signal. Short-term neurological scores or laboratory improvements alone cannot justify progression.

### 8.4 Cross-cutting research priorities

Research should first define one product and resolve its chemistry, formulation, pharmacokinetics, unbound exposure, and matched vehicle or carrier controls. Next, it should establish exposure-linked engagement of a few targets measurable in animals and patients, prioritizing endothelial barrier injury and mitochondrial stress. Durable benefit and reperfusion safety then require testing in aged, comorbid models with delayed treatment. Independent multicenter preclinical confirmation should follow completion of these stages. Ferroptosis, pyroptosis, spatial profiling, and single-cell discovery remain secondary until replicated and linked to achievable product-specific exposure.

Multicenter validation cannot resolve undefined products or unknown exposure, and pharmacokinetics alone cannot establish efficacy. Product definition and pharmacokinetics must precede target engagement, realistic efficacy testing, and multicenter confirmation. Models should reflect large-artery atherosclerosis, cardioembolism, small-vessel occlusion, incomplete reperfusion, age, sex, and common comorbidities. Data and protocol sharing should ensure that negative findings remain visible. Natural origin and historical use may support discovery but do not change modern evidentiary standards.

## 9 Conclusion

Tan IIA and STS show biological activity across oxidative and mitochondrial injury, endothelial barrier dysfunction, neuroinflammation, metabolism, regulated cell death, and repair. The parent compound, sulfonated derivative, and carrier products nevertheless have distinct exposure pathways. Endothelial barrier modulation is the most coherent cross-domain hypothesis but remains unvalidated as a mediator of clinical benefit. TLR4/NF-κB, TGM2/PANX1, ferroptosis, pyroptosis, and carrier-associated pathways remain predominantly hypothesis-generating. Incomplete product definition, weak exposure-response linkage, early dosing, young healthy animals, short surrogate outcomes, limited replication, and small heterogeneous trials constrain the evidence. The 42-patient trial and 2025 meta-analysis justify further study. They do not establish benefits for ordinal disability, activities-of-daily-living, survival, recurrent-stroke, or safety regarding uncommon bleeding.

Development should select one drug product and clinical setting, establish compartment-specific target engagement at a clinically achievable exposure, and demonstrate durable benefit in aged and comorbid reperfusion models. Independent multicenter confirmation is then required before further progression. STS is more operationally tractable for investigation, but superior efficacy remains unproven. Until these criteria are met, Tan IIA and STS should remain investigational candidates rather than standard stroke therapies.

## Glossary

ACSL4: acyl-CoA synthetase long-chain family member 4
Ang-1: angiopoietin-1
ARRIVE: Animal Research: Reporting of *In Vivo* Experiments
BBB: blood–brain barrier
CHIP: carboxyl terminus of Hsc70-interacting protein
CMC: chemistry manufacturing and controls
ECASS: European Cooperative Acute Stroke Study
EVT: endovascular thrombectomy
GFAP: glial fibrillary acidic protein
GLUT1: glucose transporter 1
HMGB1: high-mobility group box 1
KLF4: Krüppel-like factor 4
LC–MS/MS: liquid chromatography–tandem mass spectrometry
MCAO: middle cerebral artery occlusion
MCAO/R: middle cerebral artery occlusion/reperfusion
MMP-9: matrix metalloproteinase-9
mRS: modified Rankin Scale
NF-κB: nuclear factor kappa B
NIHSS: National Institutes of Health Stroke Scale
NLRP3: NLR family pyrin domain containing 3
OGD/R: oxygen–glucose deprivation/reoxygenation
PANX1: pannexin 1
PI3K/Akt/mTOR: phosphoinositide 3-kinase/protein kinase B/mechanistic target of rapamycin
PK/PD: pharmacokinetics/pharmacodynamics
PRIMED2: Preclinical evidence of Readiness In stroke Models Evaluating Drugs and Devices
rt-PA: recombinant tissue plasminogen activator
sICH: symptomatic intracranial hemorrhage
SITS-MOST: Safe Implementation of Thrombolysis in Stroke-Monitoring Study
SPAN: Stroke Preclinical Assessment Network
STAIR: Stroke Therapy Academic Industry Roundtable
STS: sodium tanshinone IIA sulfonate
Tan IIA: tanshinone IIA
TGM2: transglutaminase 2
TIMP-1: tissue inhibitor of metalloproteinase 1
TUNEL: terminal deoxynucleotidyl transferase dUTP nick-end labeling
VEGF: vascular endothelial growth factor
VEGFR2: vascular endothelial growth factor receptor 2
ZO-1: zonula occludens-1
