# Supplementary material for: Tanshinone IIA and sodium tanshinone IIA sulfonate in ischemic stroke: evidence, exposure, and translational readiness
Source: Front Mol Neurosci. 2026 Sep 23;19:1959038. doi: 10.3389/fnmol.2026.1959038 (PMC13643747; doi:10.3389/fnmol.2026.1959038)
Supplement: Supplementary file 1 [file Data_Sheet_1.PDF]

Supplementary Tables S1-S4

Reference numbers correspond to the reference list in the main manuscript. All confidence categories are author-assessed qualitative labels rather than formal GRADE or PRIMED² ratings.

Supplementary Table S1. Source-verified product-specific matrix of key preclinical and human evidence

The matrix prioritizes studies that are most informative for product identity, treatment timing, translational design, clinical signal, or large-animal feasibility and for which decision-relevant details were verified from the primary report. It is not an exhaustive inventory of every mechanism cited in the review; other emerging mechanisms are summarized in the main text and Supplementary Table S4. "Not reported in the source article" is used only when the primary report did not provide the information, and "not applicable" is used for domains that do not apply.

Study	Entity / product	Model / population	Dose and route	First dose / timing	Follow-up / main outcome	Design / reporting indicators	Main result	Exposure / target evidence	Principal translational limitation
Tang et al., 2010 [18]	Tan IIA (>99%; DMSO vehicle)	Sprague-Dawley rats, 250±20 g; 2-h transient MCAO	10, 20, or 30 mg/kg intraperitoneally; vehicle control	5 min after surgery/MCAO	24 h; infarct area, water content, Evans blue leakage, ICAM-1, MMP-9, ZO-1, occludin	Random allocation to five groups; n=6 per reported outcome; blinding and sample-size calculation not reported in the source article	Dose-related reductions in BBB leakage and MMP-9/ICAM-1 with preservation of ZO-1/occludin; 10 mg/kg did not significantly reduce water content	No plasma or unbound brain PK; pathway markers were not linked to measured exposure	Very early dosing, short follow-up, young healthy rats, and no modern reperfusion-interaction study
Chen et al., 2012 [36]	Tan IIA (99%; PBS/1% DMSO vehicle)	Adult male Sprague-Dawley rats, 250±20 g, 90±4 days; 2-h MCAO/reperfusion; 132 enrolled	25 mg/kg intraperitoneally	10 min after MCAO	6 h after reperfusion for injury outcomes; MIF-related measures at 1, 3, 6, and 24 h	Randomized, 44 per main group; blinded neurological and infarct assessment; 14/132 animals excluded or died; assay-specific n=5-8	Reduced infarct burden, edema, neurological deficit, apoptosis, and MIF-associated inflammatory signaling	No plasma or unbound brain exposure; target modulation was not linked to concentration	Early dosing, acute endpoints, male healthy rats, and no independent or clinically realistic reperfusion validation
Ji et al., 2017 [35]	STS injection	Adults with acute ischemic stroke treated with intravenous rt-PA; randomized single-center trial; 46 participants enrolled	STS 60 mg/day intravenously for 10 days vs equivalent-volume saline placebo	After rt-PA; exact onset-to-first-STS interval not reported in the article	90-day dichotomized mRS; day-10 clinical, imaging, and biomarker outcomes; 42 participants included in clinical/prognostic analysis (21/21), with an imaging/biomarker subset of 30	Randomized, double-blind, placebo-controlled, single center; four participants not in final analysis; multiple outcomes and subset analyses	mRS≤1: 16/21 vs 9/21 (RR 1.78, 95% CI 1.03-3.08); no significant day-10 NIHSS, ADL, or infarct-volume difference; hemorrhagic transformation 2/21 vs 5/21	No measured plasma exposure-target-response or mediation analysis; barrier signals were surrogate subset outcomes	Underpowered for efficacy and uncommon bleeding; dichotomized mRS, multiplicity, attrition, and no independent replication
Song et al., 2021 [37]	Tan IIA	Adult male Sprague-Dawley rats (~250 g); MCAO/reperfusion; BV2 OGD model	1, 3, or 9 mg/kg by tail vein in vivo; 10 μM in vitro	10 min after ischemia and again after reperfusion; in vitro pretreatment before 4-h OGD	24 h; infarct/neurological outcomes and microglial activation/polarization markers	Randomized six groups, 12 rats/group; n=6 for infarct analysis; three deaths reported; sample-size calculation and allocation concealment not reported	3 and 9 mg/kg reduced infarct burden and shifted NF-κB-associated microglial markers	No plasma or brain PK; 10 μM cell exposure not bridged to clinical concentration	Binary M1/M2 framework, early dosing, short follow-up, male healthy rats, and no independent replication
Xu et al., 2021 [38]	STS in saline	92 adult male Sprague-Dawley rats, 7-8 weeks, 280-320 g; 2-h MCAO/72-h reperfusion	5, 15, or 30 mg/kg intraperitoneally in dose screen; 30 mg/kg selected; daily for 3 days	First dose 2 h after reperfusion	72 h; neurological score, infarct, BBB permeability, edema, apoptosis, vascular density, VEGF/VEGFR2/Ang-1/CD31	Randomized four main groups of 20; four animals per dose in screening; blinded TTC and neurological assessment; assay-specific n=3-4	30 mg/kg reduced acute injury and increased vascular and angiogenesis-related markers	No exposure-linked angiogenic response or functional vessel assessment	Only 3-day follow-up; young male rats; early permeability safety and durable vascular benefit remain uncertain
Waters et al., 2021 [30]	PLGA-PEG Tan IIA-loaded nanoparticles	Porcine ischemic-stroke model	0.133 mg/kg intracisternally (dose reported for the linked pig program)	Approximately 1 h after stroke	Acute/subacute tissue injury, hemorrhage, white-matter damage, and functional deficits	Randomized proof-of-concept large-animal study; n=2 pigs per group (4 total); invasive local delivery; inferential statistical analysis of in-vivo endpoints was limited because of the sample size	Reduced lesion-related tissue injury and functional deficits compared with vehicle-treated stroke controls	No plasma, CSF, or unbound brain exposure-target-response relationship was reported	Invasive route, small proof-of-concept study, and unresolved CMC, scale-up, and emergency-workflow feasibility
Kaiser et al., 2022 [31]	Tan IIA nanoparticles plus induced-pluripotent-stem-cell-derived neural stem cells	Porcine ischemic-stroke model; combined nanoparticle and induced-pluripotent-stem-cell-derived neural stem-cell study; 22 pigs	Tan IIA nanoparticles 0.133 mg/kg intrathecally/intrathecally into CSF; neural stem cells transplanted 5 days after stroke	Nanoparticles approximately 1 h after stroke; neural stem cells at day 5	12 weeks; lesion, engraftment, tissue, motor, and recovery outcomes	22 pigs randomized among three groups; double-blinded study with power calculation; no exclusions reported; principal 12-week analyses n=6/group	Combination treatment improved cell survival and several tissue/functional recovery measures; acute lesion reduction preceded cell transplantation	Contribution of nanoparticles and cell therapy to long-term effects could not be separated	Combination product cannot establish Tan IIA monotherapy efficacy and creates complex manufacturing/regulatory requirements

Study	Entity / product	Model / population	Dose and route	First dose / timing	Follow-up / main outcome	Design / reporting indicators	Main result	Exposure / target evidence	Principal translational limitation
Liu et al., 2025 [42]	STS injection as adjunctive therapy	Systematic review/meta-analysis of 22 randomized trials; 2,289 participants	Heterogeneous intravenous regimens	Heterogeneous acute-treatment windows	Short-term neurological, physiological, hemorheological, coagulation, lipid, biochemical, ADL, and safety outcomes; searches through Feb 2024	Source trials were generally small and regionally concentrated; intervention, background care, blinding, and outcome definitions varied	Pooled direction favored several short-term outcomes; no significant ADL improvement was established	No integrated exposure-response analysis	Does not establish ordinal disability, survival, recurrent stroke, quality of life, or uncommon bleeding safety

Supplementary Table S2. Author-assessed translational confidence and minimum next steps

Confidence categories are descriptive: Limited indicates a major unresolved prerequisite; Suggestive indicates directionally supportive but incomplete evidence; Moderately coherent indicates convergence across domains without confirmatory readiness; Operationally tractable describes product or workflow practicality, not efficacy. Judgments are informed by ARRIVE 2.0, STAIR, PRIMED², and SPAN principles [3-8,28] and are not validated numerical scores.

Domain	Author-assessed confidence	Evidence supporting the judgment	Principal gap	Minimum next step
Drug identity and product definition	Suggestive	Tan IIA and STS are chemically distinct; several carriers are described [10,21–24,29–31].	Non-equivalent products are still discussed together; final product specifications are absent.	Select one candidate and define active entity, excipients, route, release specifications, stability, and CMC.
Mechanistic plausibility	Suggestive overall	Directionally convergent findings span redox, mitochondria, BBB, inflammation, death, metabolism, and repair [10–20,32–34,36–38] ; endothelial-barrier evidence is the most coherent cross-domain hypothesis.	Many pathways are downstream, overlapping, formulation-dependent, or single-laboratory observations; product-specific exposure linkage is sparse.	Prioritize one or two measurable mechanisms with causal interruption and human-compatible markers.
Compartment-specific exposure	Limited	Human plasma STS assays and animal distribution reports exist [22–24]..	Luminal endothelial, unbound parenchymal, and peripheral exposures are not linked to outcome.	Perform stroke-specific plasma and mechanism-relevant compartment PK/PD with exposure-response modeling.
Dose-response	Limited	Several nominal doses have been used.	Matched unbound exposure and minimally effective exposure are rarely defined.	Define minimally effective and maximally tolerated exposure using the final product.
Therapeutic window	Limited	Some post-ischemic dosing studies exist.	Pretreatment, immediate dosing, and short delays remain common.	Test prespecified delayed dosing after imaging and after reperfusion.
Population/model diversity	Suggestive	Cell, rodent, and limited porcine models are available[11,12,14,16–19,29–34,36–38] .	Aging, both sexes, hypertension, diabetes, thromboembolic stroke, and treatment heterogeneity are underrepresented.	Replicate in aged animals of both sexes with common comorbidity across more than one model.
Reperfusion compatibility	Limited	One small STS-plus-rt-PA trial and coherent endothelial rationale exist [18,34,35]..	Tenecteplase/thrombectomy settings and adjudicated bleeding are rarely modeled.	Test efficacy and interactions with thrombolysis and mechanical reperfusion, including sICH and systemic bleeding.
Durable functional efficacy	Limited	Some restorative and large-animal studies report function [30,31,38].	The 12-week study used combination therapy and cannot isolate the nanoparticle effect; most other studies emphasize 24-72-hour outcomes.	Require ≥28-day multidomain functional assessment, mortality, hemorrhagic transformation, cognition when relevant, and tissue outcomes.
Methodological robustness	Suggestive	ARRIVE 2.0, STAIR, PRIMED², and SPAN provide standards [3–8,28].	Randomization, allocation concealment, blinding, sample-size planning, exclusions, and deaths are inconsistently reported.	Preregister the confirmatory protocol, prespecify analyses, report all exclusions and deaths, and use blinded centralized assessment.
Independent replication	Limited	Directional consistency exists across publications.	Specific product-dose-mechanism claims are rarely reproduced by an independent group; repetition within one laboratory is not independent replication.	Replicate the fixed product-dose hypothesis in at least two independent centers.
Human efficacy	Limited	One 42-patient trial and a 22-trial meta-analysis provide signals [35,42].	Small heterogeneous studies, regional concentration, nonstandard outcomes, and sparse ordinal mRS/ADL data.	Conduct a multicenter double-blind phase IIb trial with ordinal 90-day mRS.
Human safety	Limited	No dominant safety signal has been reported [35,42].	Power and adjudication are inadequate for sICH, systemic bleeding, reactions, and interactions.	Complete stroke-specific interaction and safety studies with central adjudication.

Domain	Author-assessed confidence	Evidence supporting the judgment	Principal gap	Minimum next step
Operational feasibility	More operationally tractable for STS; limited for advanced carriers	STS is water soluble and intravenously administrable; carriers may improve delivery [10,21,29,30].	International stroke indication, CMC, and emergency workflow remain undefined.	Define the indication, engage regulators, and test stroke-unit/reperfusion workflow compatibility.

Supplementary Table S3. Search modules and reproducibility record for the structured narrative review

The search was purpose-driven rather than designed to estimate a pooled treatment effect. The modules below were completed through 28 July 2026 and used to identify representative evidence. No PRISMA flow, dual exhaustive screening, formal study-level risk-of-bias adjudication, or de novo pooled estimate is claimed. PubMed/MEDLINE, registry searches, and citation tracking formed the core strategy; Chinese-language and non-indexed evidence may therefore be underrepresented. Exact queries, dates, and exported citations should be retained in the author archive.

Source / module	Completed search approach	Coverage in this review	Evidence contribution	Known limitation / archive requirement
PubMed/MEDLINE: stroke efficacy and mechanism	(tanshinone IIA OR tanshinone 2A OR sodium tanshinone IIA sulfonate OR tanshinone IIA sodium sulfonate) AND (ischemic stroke OR cerebral ischemia OR MCAO OR ischemia-reperfusion OR oxygen-glucose deprivation)	Inception through 28 July 2026; human, animal, and in vitro records	Core mechanistic, rodent, cell, and recent pathway reports	Purpose-driven selection, not exhaustive screening; retain exact final query/date/export.
PubMed/MEDLINE: pharmacokinetics and distribution	Compound block AND (pharmacokinetic* OR biodistribution OR brain penetration OR blood-brain barrier OR LC-MS/MS OR plasma OR unbound)	Inception through 28 July 2026; stroke and non-stroke PK used when relevant	Human plasma assay/PK, animal distribution, and efflux evidence	Non-stroke PK may not represent acute stroke; retain product, dose, and population details.
PubMed/MEDLINE: formulation and CMC	Compound block AND (liposome OR nanoparticle OR nanoliposome OR borneol OR formulation OR delivery OR stability OR manufacturing)	Inception through 28 July 2026	Carrier and large-animal delivery studies	Search identifies feasibility signals, not regulatory qualification; record carrier composition and route.
PubMed/MEDLINE: human clinical evidence	Compound block AND (stroke OR cerebral infarction) AND (random* OR trial OR clinical OR meta-analysis OR systematic review)	Inception through 28 July 2026	2017 controlled study and 2025 evidence synthesis	Regional/non-English reports may be incompletely indexed; verify duplicate cohorts and outcome definitions.
ClinicalTrials.gov	tanshinone; tanshinone IIA; sodium tanshinone IIA sulfonate; condition terms stroke/cerebral infarction	All statuses searched through 28 July 2026	Screening for active, completed, or unpublished programs	Negative search is term- and registry-dependent; retain date, terms, and result export.
Chinese Clinical Trial Registry	tanshinone; tanshinone IIA; sodium tanshinone IIA sulfonate; stroke; ChiCTR-ONRC-14004659	Registry verification through 28 July 2026	Registration identifier associated with the 2017 trial; prospective status was not independently confirmed.	Avoid describing the trial as prospectively registered unless the registry date and first enrollment are verified.
Reference-list and citation chasing	Backward screening of reviews and primary reports; forward tracking of clinical, PK, large-animal, and key mechanistic studies	Principal source papers and linked records	Studies missed by keyword indexing and later related reports	Record the parent citation through which each additional study was identified.
Coverage statement	No PRISMA flow, dual exhaustive screening, or de novo pooling was performed	Structured critical narrative review	Supports decision-focused synthesis	Do not describe the dataset as exhaustive or the confidence judgments as formal GRADE/PRIMED² scores.

Supplementary Table S4. Outcome-level author-assessed confidence and permitted interpretation

Confidence categories are qualitative and are not formal GRADE ratings because complete study-level risk-of-bias adjudication was not performed for every report. “Limited” means that the current evidence cannot support a clinical claim; “suggestive” denotes a hypothesis-generating signal. The final column states the strongest interpretation permitted by the available evidence.

Outcome / question	Available evidence	Main limitations	Author-assessed confidence	Strongest permitted interpretation
90-day functional disability (mRS)	One placebo-controlled STS-plus-rt-PA trial: mRS≤1 at 90 days in 16/21 vs 9/21; sparse pooled hard-outcome data [35, 42] .	42 analyzed participants; RR 1.78 (95% CI 1.03-3.08), but mRS was dichotomized; multiplicity and no independent replication.	Suggestive signal; limited clinical confidence	A favorable hypothesis-generating signal exists, but reduction in disability is not established.
Activities of daily living	Day-10 ADL 82.62±20.89 vs 72.38±25.18 (mean difference 10.24, approximate 95% CI -3.75 to 24.23; P=0.159); meta-analysis reported no significant ADL improvement [35, 42] .	Short follow-up, small sample, heterogeneous measurement, and sparse reporting.	Limited	No convincing improvement in activities of daily living has been established.

Outcome / question	Available evidence	Main limitations	Author-assessed confidence	Strongest permitted interpretation
Early neurological deficit (NIHSS or similar)	Several small trials and the 2025 meta-analysis suggest improvement [42].	Dose, timing, background care, blinding, clinical importance, and reporting are heterogeneous.	Suggestive	STS may affect short-term neurological scores, but magnitude and patient relevance are uncertain.
Mortality	Limited and inconsistent reporting.	Trials are underpowered and follow-up is often short.	Limited	No reliable mortality benefit can be inferred.
Hemorrhagic transformation and major bleeding safety	Hemorrhagic transformation 2/21 vs 5/21 in the controlled study (RR 0.40, 95% CI 0.09-1.84; P=0.410), plus limited pooled safety reporting [35, 42]	Rare events, very wide confidence interval, incomplete adjudication, and limited interaction testing.	Limited	Absence of a detected signal does not establish hemorrhagic safety or compatibility with contemporary reperfusion care.
Recurrent stroke / vascular events	Little robust long-term randomized evidence.	Short follow-up and surrogate focus.	Limited	No secondary-prevention conclusion is justified.
BBB permeability and related biomarkers	Human CT-perfusion/biomarker signal plus Tan IIA endothelial and rodent findings [18, 34, 35]	Small subset; cross-entity inference; surrogate outcomes; no mediation or validated exposure-target-response link.	Moderately coherent biological hypothesis; limited clinical mediation confidence	Barrier modulation may support target-engagement studies but cannot replace patient-centered outcomes.
Preclinical infarct volume / early scores	Directionally consistent experimental findings [10–20, 32, 36–38].	Young healthy animals, early dosing, variable products, incomplete reporting, and limited independent replication.	Suggestive biological activity; limited clinical prediction	Experimental activity is plausible, but human efficacy cannot be inferred.
Long-term preclinical function	One 12-week porcine combination-treatment study reports sustained functional recovery [31]; other cited product-specific studies are acute/subacute [30, 38].	The durable study used combination therapy and cannot isolate the Tan IIA nanoparticle effect; evidence is limited by invasive delivery, limited comorbidity, and no multicenter confirmation.	Suggestive	Durable recovery remains unestablished for a fixed clinically deployable product.
Emerging pathways	TLR4/NF-κB, TGM2/PANX1, miR-449a/ACSL4, KLF4/CHIP/NLRP3, and carrier-associated PGK1/Nrf2 reports[10, 13–15, 20].	Mostly single-laboratory evidence with limited causal replication, exposure linkage, and human validation.	Limited	These pathways are hypothesis-generating and should not determine clinical progression alone.

Abbreviations: ADL, activities of daily living; BBB, blood-brain barrier; CMC, chemistry, manufacturing and controls; CT, computed tomography; LC-MS/MS, liquid chromatography-tandem mass spectrometry; MCAO, middle cerebral artery occlusion; MCAO/R, middle cerebral artery occlusion/reperfusion; mRS, modified Rankin Scale; NIHSS, National Institutes of Health Stroke Scale; OGD, oxygen-glucose deprivation; PK/PD, pharmacokinetics/pharmacodynamics; PRIMED², Preclinical evidence of Readiness In stroke Models Evaluating Drugs and Devices; rt-PA, recombinant tissue plasminogen activator; sICH, symptomatic intracranial hemorrhage; SPAN, Stroke Preclinical Assessment Network; STAIR, Stroke Therapy Academic Industry Roundtable; STS, sodium tanshinone IIA sulfonate; Tan IIA, tanshinone IIA.
